# The Role of Extracellular Vesicles in β-Cell Function and Viability: A Scoping Review

**DOI:** 10.3389/fendo.2020.00375

**Published:** 2020-06-11

**Authors:** Stephanie Chidester, Alicia A. Livinski, Anne F. Fish, Paule V. Joseph

**Affiliations:** ^1^Sensory Science & Metabolism Unit, Biobehavioral Branch, National Institute of Nursing Research, Division of Intramural Research, National Institutes of Health, Department of Health and Human Services, Bethesda, MD, United States; ^2^College of Nursing, University of Missouri-St. Louis, St. Louis, MO, United States; ^3^National Institutes of Health Library, Office of Research Services, OD, Department of Health and Human Services, National Institutes of Health, Bethesda, MD, United States

**Keywords:** β-cell, diabetes, extracellular vesicle, exosome, insulin secretion

## Abstract

Extracellular vesicles (EVs) released by cells throughout the body have been implicated in diabetes pathogenesis. Understanding the role of EVs in regulation of β-cell function and viability may provide insights into diabetes etiology and may lead to the development of more effective screening and diagnostic tools to detect diabetes earlier and prevent disease progression. This review was conducted to determine what is known from the literature about the effect of EV crosstalk on pancreatic β-cell function and viability in the pathogenesis of diabetes mellitus, to perform a gap analysis for future research directions, and to discuss implications of available evidence for diabetes care. The literature search yielded 380 studies from which 31 studies were determined to meet eligibility criteria. The majority of studies had the disease context of autoimmunity in T1DM. The most commonly studied EV crosstalk dynamics involved localized EV-mediated communication between β-cells and other islet cells, or between β-cells and immune cells. Other organs and tissues secreting EVs that affect β-cells include skeletal muscle, hepatocytes, adipocytes, immune cells, bone marrow, vascular endothelium, and mesenchymal stem cells. Characterization of EV cargo molecules with regulatory effects in β-cells was conducted in 24 studies, with primary focus on microRNA cargo. Gaps identified included scarcity of evidence for the effect on β-cell function and viability of EVs from major metabolic organs/tissues such as muscle, liver, and adipose depots. Future research should address these gaps as well as characterize a broader range of EV cargo molecules and their activity in β-cells.

## Introduction

Diabetes has reached epidemic proportions both globally and in the United States (1, 2). Type 1 diabetes mellitus (T1DM) and type 2 diabetes mellitus (T2DM) affect ~34.2 million individuals in the United States, 13% of the adult population (2). Worldwide, diabetes prevalence has reached 8.5%, affecting more than 422 million people (1). During 2019, gestational diabetes (GDM) affected an estimated 17.05 million (13.2%) pregnancies resulting in live births worldwide. Although this type of diabetes is usually transitory and most cases resolve after delivery, women who experience GDM are 50% more likely to develop T2DM subsequently, and children exposed to GDM *in utero* are at increased risk for obesity and T2DM (3). Many cases of diabetes are not diagnosed until disease progression is advanced and complications are beginning to manifest (1, 2, 4). There is a critical need for earlier and more effective screening and diagnostic tools, followed by personalized interventions to prevent disease progression of diabetes.

A common feature of T1DM, T2DM, and GDM pathogenesis is impairment of insulin secretion capacity (5, 6). In T1DM, this impairment typically occurs due to autoimmune targeting of β-cells within pancreatic islets and subsequent depletion of islet β-cell mass (5). In T2DM and GDM, this impairment occurs in the setting of systemic insulin resistance, leading initially to hypertrophy and proliferation of pancreatic β-cells in order to increase insulin secretion capacity (5–7). As disease severity progresses, β-cells become progressively more dysfunctional and begin to fail, resulting in inadequate insulin secretion and elevated blood glucose levels (5–7). In advanced T2DM, populations of β-cells may undergo de-differentiation and/or apoptosis (5, 7). Symptom onset in diabetes mellitus typically coincides with a significant decrease in the quantity or functionality of islet β-cells.

**Figure 1:**
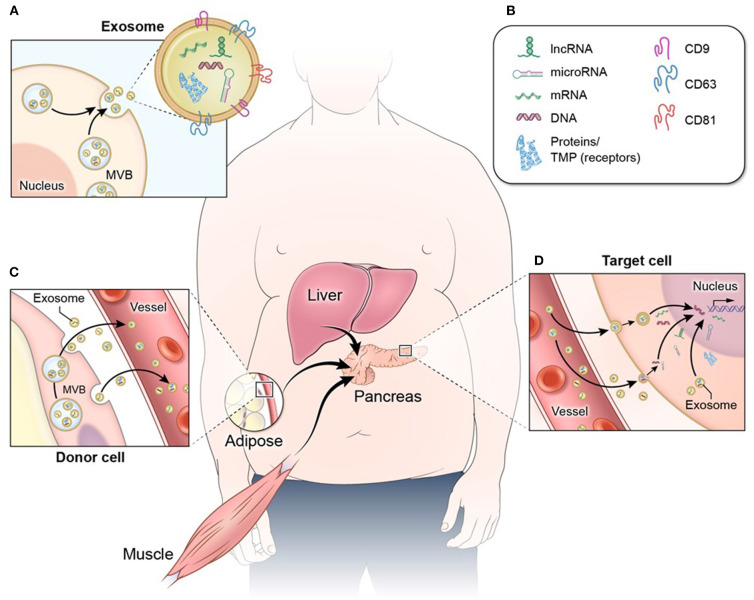
Exosome structure and function. **(A)** Exosomes and other EVs consist of a protective lipid bilayer membrane with transmembrane and surface receptor proteins. **(A,B)** This membrane encloses a diverse bioactive cargo of proteins, non-coding RNA (including long non-coding RNA and microRNA), mRNA, and DNA fragments. **(C)** EVs released from metabolic tissues and organs such as adipose, muscle, and liver enter circulation and **(D)** migrate to distal tissues such as pancreatic islets, where they are internalized by target cells. When EV cargo is released into a target cell, the proteins and RNA species can have a number of effects that include cell signaling cascades and regulation of gene expression.

Declining β-cell function and/or mass are the result of complex crosstalk between pancreatic islets and other tissues throughout the body (8, 9). This crosstalk is mediated in part by extracellular vesicles (EVs), including exosomes, microvesicles, and apoptotic bodies. Exosomes are EVs of ~50–150 nm in diameter that are secreted by cells throughout the body and convey complex molecular messages to other cells in order to coordinate metabolic function (10–13). These EVs originate within the cell, inside endosomes, and they consist of a lipid bilayer membrane with embedded protein molecules and an inner lumen containing a diverse cargo of lipid, protein, and nucleic acid species (13, 14) (Figure 1). Microvesicles are similar in structure, content, and function to exosomes, but are larger in diameter (100–1,000 nm) and are formed at the plasma membrane by budding (10–13). Apoptotic bodies are formed in the process of cell death from fragments of the parent cell. They range widely in size (100–5,000 nm in diameter), and the lipid bilayer membrane may enclose cellular organelles as well as lipids, proteins, and nucleic acids (13, 15). When released from cells, EVs may interact with nearby cells or migrate through the bloodstream to cells in distal organs and tissues (16, 17) (Figure 1). EVs and their bioactive cargo can significantly impact the capacity of pancreatic β-cells to produce and secrete insulin, and they may also impact β-cell survival through EV cargo that affect proliferative, inflammatory, or apoptotic pathways (18–20). Because of the practical difficulties inherent in distinguishing exosomes from other small EVs in a biofluid, we use the abbreviated terms “small EVs” for vesicles consistent with characteristics of exosomes and small microvesicles and “large EVs” for mixed vesicle populations of microvesicles and apoptotic bodies, in acknowledgment that samples of EVs described in the research literature as exosomes or microvesicles may include other vesicles of similar size (21, 22).

This scoping review examines the evidence for the effect of EV crosstalk on β-cell viability and function, within the context of T1DM, T2DM, and GDM. For the purposes of this review, β-cell viability refers to cell survival or death, and function encompasses aspects of cell differentiation, changes in cellular phenotype, glucose sensing, and insulin production and secretion. The aims of this review are: (a) to determine what is known from the literature to date about the effect of EV crosstalk on pancreatic β-cell function and viability in diabetes mellitus, (b) to perform a gap analysis for future research directions, and (c) to describe implications of this evidence for aspects of diabetes care, including diagnosis, delay of onset, and treatment.

## Methods

An *a priori* protocol for a scoping review of research literature was created (Supplementary Methods 1), based on the framework described by Arksey and O'Malley (23).

### Identification of Relevant Studies

The inclusion criteria were: (a) original research with an experimental design, (b) use of an *in vivo, ex vivo*, or *in vitro* model of β-cell function, (c) experimental intervention involving EVs or their cargo, (d) one or more outcome measures of β-cell function or viability, and (e) disease context of T1DM, T2DM, or GDM. Studies were required to be available in the English language. Sources of evidence were excluded if they were not original research studies or used a non-experimental design, including reviews, editorials, opinion articles, and letters. Also excluded were studies involving vesicles that had been modified or engineered.

To identify relevant studies, literature searches were conducted (AAL and SC) using the following databases: Cochrane Library: Database of Systematic Reviews (Wiley); Cochrane Library: CENTRAL (Wiley); Embase (Elsevier); PubMed/MEDLINE (National Library of Medicine); Scopus (Elsevier); and Web of Science: Core Collection (Clarivate Analytics). The Dissertations & Theses Global (ProQuest) database was also searched. The literature searches were conducted between January 11, 2019 and April 8, 2020. Additionally, bibliographies of relevant research and review articles were manually searched to identify additional studies that were not already retrieved with the database searches.

A combination of keywords and controlled vocabulary terms were used to conduct the database searches. For PubMed/MEDLINE and Cochrane Library searches, the Medical Subject Headings (MeSH) and in Embase the EMTREE controlled vocabulary terms were used. The terms used described each part of the research question and included the following in addition to other synonyms: exosome, extracellular vesicle, microvesicle, nanovesicle, insulin-secreting cells, pancreatic β-cells, β-cell function, diabetes mellitus, prediabetes, insulin resistance, glucose intolerance, obesity, and metabolic syndrome. Searches were limited to English language only, but no publication date or article type limits were used. Detailed search strategies are provided in Supplementary Methods 2.

### Study Selection

Database search results were compiled in an EndNote library (Clarivate Analytics, version X9.1). Duplicate records were identified and removed, and the remaining unique results were independently reviewed (SC and PVJ) for eligibility. EndNote was used for the initial title/abstract review of the results, as well as the full-text review of potentially eligible studies. After independent selection of studies for inclusion, the results were compared and discussed (SC and PVJ). An arbiter (AFF) was designated to adjudicate any disagreements in study selection.

### Data Charting

The following data and concepts were extracted from studies that met inclusion criteria: author(s), year of publication, disease context, model system, EV crosstalk dynamics, EV cargo molecules implicated, β-cell outcome measures, and therapeutic implications. Data charting was conducted independently (SC).

### Collating, Summarizing, and Reporting Results

Extracted data and concepts were examined and organized in order to determine the extent of the evidence available for this topic, identify trends, and facilitate gap analysis (SC). Results were summarized and presented in tables and figures (SC).

## Results

**Figure 2:**
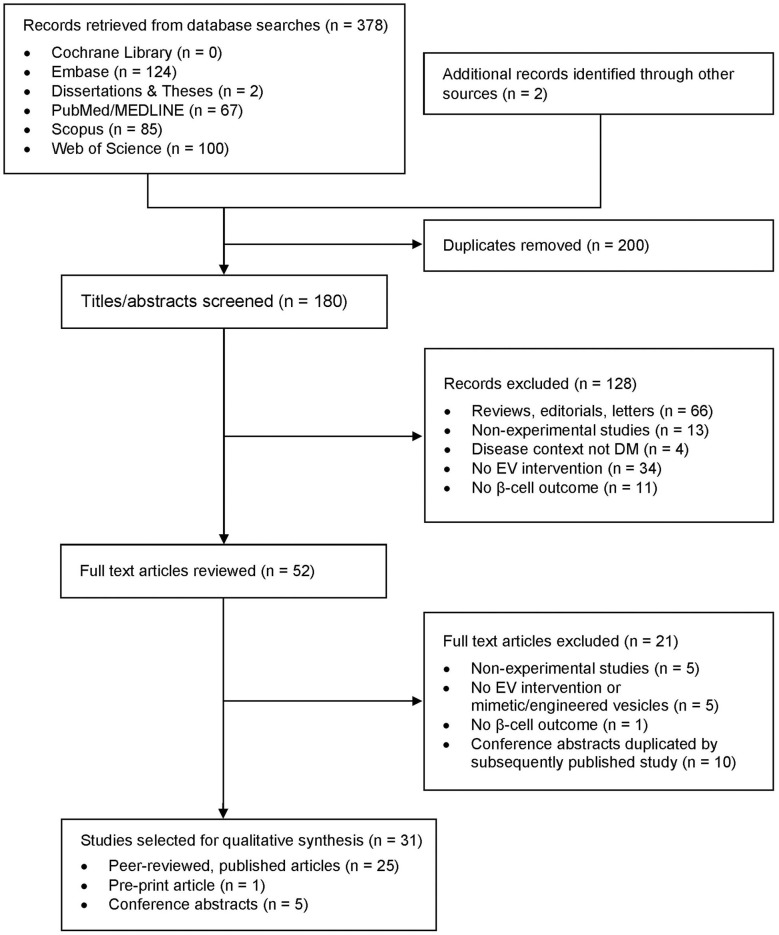
Flow diagram showing study selection process.

The database searches yielded 378 results, including publications found in the latest search (Figure 2). Two additional records were identified through the manual search of bibliographies. After duplicate records were removed, 180 unique results were identified. Title and abstract screening for inclusion and exclusion criteria led to the exclusion of 128 records. Full text review of the remaining 52 records resulted in the selection of 31 studies for analysis in this review: 25 peer-reviewed, published articles and 6 studies from the gray literature, including 1 pre-print article and 5 conference abstracts (Table 1).

**Table 1:** Summary of studies selected for review.

**First author, (ref)**	**Model, conditions**	**EV-mediated β-cell outcome**	**Therapeutic implications**
**β-cell (or islet)/β-cell EV crosstalk**			
Guay et al. (24)	T1DM and T2DM; *ex vivo* (islets from Wistar rat); *in vitro* (MIN6-B1*, INS-1* cell lines) • Pro-inflammatory cytokines	Viability: • Transmission of pro-apoptotic signals to other β-cells through EV-mediated transfer of miRNAs (cytokine exposure)	Implicated miRNAs are potential therapeutic targets (anti-miRNA therapies)
Ribeiro et al. (25)	T2DM; *ex vivo* (human islets*); *in vitro* (amyloid aggregation) • Amyloid toxicity	Viability (indirect, secondary to IAPP amyloid toxicity): • Reduced amyloid aggregation (treatment with EVs from islets of healthy donors) • Increased amyloid aggregation (treatment with EVs from islets of T2DM donors) • Altered ratio of lipids to proteins in EVs from T2DM donors	Potential for use of engineered lipid vesicles in prevention of IAPP amyloid deposits
Ruan et al. (26)	T2DM; *ex vivo* (human serum*; islets from db/db, db/m mice); *in vitro* (MIN6 cells*) • Hyperglycemia	Viability and function: • Protection from apoptosis via EV-mediated transfer of lncRNA-p3134 (hyperglycemic conditions) • Increased insulin synthesis and secretion	Potential use of lncRNA-p3134 in development of lncRNA-based therapeutics for induction of compensatory insulin secretion
Tang et al. (27)	T2DM; *in vitro* (INS-1 cells*) • Lipotoxicity	Viability and function: • Protection from palmitate-induced apoptosis via EV-mediated transfer of NCDase • Preservation of glucose-sensing mechanisms	NCDase or biomimetic molecules are potential therapies to prevent lipid-induced β-cell apoptosis
Sun et al. (28)	T1DM and T2DM; *in vivo* (B6 mice), *in vitro* (MIN6-B1*, INS-1* cell lines) STZ-induced DM (low-dose and high-dose models) • Lipotoxicity	Viability and function: • Protection from palmitate-induced apoptosis (*in vitro*, treatment with EVs compared to control) • Low-dose STZ model (treatment with EVs compared to control): Increased angiogenesis within islets, improved glucose tolerance, increased insulin synthesis and secretion; decreased macrophage infiltration of islets	Characterization of EV cargo may lead to identification of therapeutic targets
Zhu et al. (29)	T1DM; *ex vivo* (islets from SD rats); *in vitro* (INS-1 cells*) • Pro-inflammatory cytokines	Viability: • Protection from cytokine-induced apoptosis (cytokine exposure, low concentration) • Increased apoptosis with loss of EV NCDase cargo (cytokine exposure, high concentration)	NCDase or biomimetic molecules are potential therapies to prevent inflammation-induced β-cell apoptosis
*Javeed et al. (30)*	*T1DM and T2DM; ex vivo (islets from mice [unspecified])*; in vitro (MIN6,* INS-1 cells*) • *Pro-inflammatory cytokines*	*Function:* • *Decreased insulin synthesis and secretion, gene expression profile associated with β-cell failure (islets treated with EVs from cytokine-exposed MIN6, compared to control EVs)* • *Increased production of exosome-sized EVs and elevated EV cargo of proteins associated with inflammation (cytokine-exposed cells, compared to control-treated MIN6, INS-1 cells)*	*Identification of EV biomarkers of β-cell failure, potential therapeutic targets*
*Sims et al. (31)*	T1DM; ex vivo (islets and serum* from NOD mice); in vitro (INS-1 828/13, 828/33 cells*) • *Autoimmunity* • *Pro-inflammatory cytokines*	*Viability:* • *Decreased β-cell mass, increased apoptosis via action of EV transfer of miR-21 (cytokine exposure)* • *Cytokine-induced increase in miR-21 cargo*	*Implicated miRNA is a potential therapeutic target (anti-miRNA therapy) for T1DM*
**β-cell/immune cell EV crosstalk**			
Bashratyan et al. (32)	T1DM; *in vivo* (NOD, B6 mice); *in vitro* (MIN6 cells*) • Autoimmunity	Viability (indirect, via activation of autoimmune response): • Activation of B lymphocytes as part of autoimmune response in T1DM (treatment with insulinoma-derived EVs) • Elevated number of EV-reactive B lymphocytes in T1DM-prone NOD mice vs. control (B6) mice • Reduced B lymphocyte reactivity to EVs (treatment with calcineurin inhibitor)	Identification of markers for B-lymphocyte reactivity to β-cell derived EVs and for predisposition to autoimmune response in T1DM
Cianciaruso et al. (33)	T1DM; *ex vivo* (islets* from human donors, SD rats, NOD mice); *in vitro* (INS-1E cells*, MIN6 cells) • Autoimmunity • Pro-inflammatory cytokines	Viability (indirect, via activation of autoimmune response): • Activation of antigen-presenting cells derived from NOD mice (treatment with islet EVs) • Islet auto-antigens present in EVs from islets and β-cell lines • Increased islet EV output (cytokine exposure)	Elevation in circulating islet-derived EVs may increase risk for activation of autoimmune response Elevated islet EV secretion may be an early indicator of T1DM development
Guay et al. (34)	T1DM; *in vivo* (NOD mice), *ex vivo* (mouse islets, T-lymphocytes*) • Autoimmunity	Viability: • β-cell mass depletion • Transfer of pro-apoptotic miRNAs from T lymphocytes to β-cells	Implicated miRNAs are potential therapeutic targets (anti-miRNA therapies)
Salama et al. (35)	T1DM; *in vivo* (NOD/ShiLtJ mice; CL4-TCR^+^Thy1.1^+^ mice); *ex vivo* (NOD splenocytes); *in vitro* (MIN6 cells*) • Autoimmunity	Viability (indirect, via activation of autoimmune response): • Induction of pro-inflammatory cytokines in NOD splenocytes via EV-mediated transfer of miR-29b (treatment with EVs from β-cell culture)	miR-29b is a potential therapeutic target (anti-miRNA therapy) for autoimmune destruction of β-cells Elevations in circulating β-cell derived EVs may increase risk for T1DM development
Sheng et al. (36)	T1DM; *in vivo* (NOD, NOR, SCID, B6 mice); *ex vivo* (pancreatic lymph nodes); *in vitro* (MIN6 cells*) • Autoimmunity	Viability (indirect, via activation of autoimmune response): • Activation of T lymphocytes; increased pro-inflammatory cytokine expression (treatment with EVs from β-cell culture) • Elevated number of reactive Th1 cells in pancreatic lymph nodes (NOD mice)	β-cell derived EVs may provoke inflammatory response and activate autoimmune response
*Giri et al. (37)*	T1DM; ex vivo (NOD/ShiLtJ mouse bone marrow-derived dendritic cells); in vitro (MIN6 cells,* RAW264.7 cells) • *Autoimmunity* • *Pro-inflammatory cytokines* • *Hypoxia*	*Viability (indirect, via activation of autoimmune response):* • *Increased activation of macrophages (treatment with large and small EVs from cytokine-exposed vs. control-treated MIN6 cells)* • *Increased activation of dendritic cells (treatment with apoptotic bodies and small EVs from cytokine-exposed vs. control treated MIN6)* • *Increased release of small EVs and apoptotic bodies containing cargo of insulin and pro-insulin autoantigens and diabetogenic miRNAs (EVs from cytokine-exposed MIN6 cells compared to control-treated cells)*	*Identification of EV markers for cytokine-induced β-cell stress and risk for activation of autoimmune response*
**Mesenchymal stem cell (MSC)/β-cell EV crosstalk**			
Mahdipour et al. (38)	T1DM; *in vivo* (Wistar rat); *ex vivo* (human MSCs* derived from menstrual blood) • STZ-induced DM • Inflammation	Viability and function: • Regeneration of β-cell mass • Increased insulin synthesis/secretion	Potential use of donor MSC-EVs as therapeutics for regeneration of functional islet β-cells
Sun et al. (39)	T2DM; *in vivo* (SD rat, HFD); *ex vivo* (human MSCs* derived from umbilical cord) • STZ-induced DM • Hyperglycemia • Insulin resistance • Lipotoxicity	Viability and function: • Increased β-cell proliferation, islet regeneration • Increased insulin secretion • Decreased circulating pro-inflammatory cytokines	Potential use of donor MSC-EVs as therapeutics for regeneration of functional islet β-cells
Tan et al. (40)	T1DM; *ex vivo* (islets from porcine neonates; MSCs* derived from human umbilical cord) • Hypoxia • Oxidative stress	Viability: • Alleviation of hypoxic β-cell injury and cell death • Decreased markers of oxidative stress in islet cell clusters	Potential use of donor MSC-EVs as therapeutics to enhance viability of islet transplants
Chen et al. (41)	T1DM; *ex vivo* (human MSCs* derived from umbilical cord); *in vitro* (βTC-6 cells) • Hypoxia	Viability: • Alleviation of hypoxia-induced endoplasmic reticulum stress and apoptosis (β-cell line treated with MSC-EVs vs. control) • Decreased expression in treated β-cells of apoptosis and ER-stress proteins • Effects attributed to MSC-EV cargo enrichment of miR-21, validated by β-cell treatment with miR-21 mimic vs. inhibitor	Potential use of donor MSC-EVs or miR-21 mimics as therapeutics to enhance viability of islet transplants
Nojehdehi et al. (42)	T1DM; *in vivo* (B6 mice); *ex vivo* (splenic mononuclear cells; adipose MSCs*) • STZ-induced DM	Viability (indirect, via suppression of autoimmune processes): • Decreased autoimmune destruction of β-cells (IP injection of EVs from adipose MSCs) • Increased expression of anti-inflammatory cytokines and decreased expression of pro-inflammatory cytokines in splenic mononuclear cells	Potential use of MSC-EVs from donor adipose tissue as therapeutics for T1DM
*Favaro et al. (43)*	T1DM and T2DM; ex vivo (MSCs* derived from human adipose tissue, PBMCs)	*Viability (indirect, via activation of immune cells, inflammatory response):* • *Elevated cargo of inflammatory cytokines in adipose MSC-EVs (healthy donors)* • *Increased PBMC expression of IFN-γ (T1DM donors)* • *Increased PBMC expression of IFN-γ, IL-6, IL-10, IL-17, TNF-α, IL-1-β (T1DM and T2DM donors)*	
**Bone marrow/β-cell EV crosstalk**			
Tsukita et al. (44)	T1DM and T2DM; *in vivo* (B6 mice); *ex vivo* (mouse islets, bone marrow cells*) • STZ-induced DM • Hyperglycemia	Viability and function: • Increased functional β-cell mass (treatment with bone marrow EVs or with miR-106b, miR-222-3p) • Reduced β-cell expression of Cip/Kip • Improved glycemic control	Implicated miRNAs or biomimetics are potential therapeutics for enhancing β-cell viability and insulin secretion capacity
Li et al. (45)	T2DM; *in vivo* (SD rat); *ex vivo* (rat bone marrow*) • STZ-induced DM • Liraglutide therapy • Ovariectomy	Viability and function: • Increased β-cell proliferation (liraglutide treatment) via EV-mediated transfer of cargo miRNAs involved with Wnt signaling pathway • Increased insulin secretion, improved glycemic control via EV-mediated transfer of cargo miRNAs involved with insulin secretion/signaling pathways	Implicated miRNAs provide insights into potential miRNA-based therapeutics for T2DM Liraglutide treatment induces bone marrow-β-cell EV crosstalk that enhances functional β-cell mass
**β-cell (or islet)/endothelium EV crosstalk**			
Cantaluppi et al. (46)	T1DM; *in vivo* (SCID mice); *ex vivo* (endothelial progenitor cells* derived from human PBMCs; human islets); *in vitro* (primary culture human islet endothelial cells)	Viability and function: • Increased β-cell survival, decreased apoptosis of cultured islets and islet xenografts (treatment with EVs [exosomes and microvesicles] from endothelial progenitor cells) • Improved glucose-stimulated insulin secretion (treatment with EVs from endothelial progenitor cells) • Promotion of angiogenesis in islet xenografts and cultured islet endothelial cells (treatment with EVs from endothelial progenitor cells)	Treatment with EVs derived from endothelial progenitor cells may improve success of islet transplants as treatment for T1DM
Figliolini et al. (47)	T1DM; *ex vivo* (islets* from human donors; *in vitro* (primary culture human islet endothelial cells)	Viability (indirect effect): • Enhancement and maintenance of islet/β-cell vascular supply • Decreased rates of apoptosis; increased rates of proliferation; increased formation of capillary-like structures in cultured islet endothelial cells (treatment with islet-derived EVs [exosomes and microvesicles predominantly of β-cell origin] compared to control treatment)	EVs released by islets of healthy donors contain factors that help maintain adequate islet vasculature. Implicated miRNA or biomimetic may have therapeutic potential for improving success of islet transplants as treatment for T1DM
**Skeletal muscle/β-cell EV crosstalk**			
Jalabert et al. (48)	T2DM; *in vivo* (B6 mice, HPD vs SCD); *ex vivo* (mouse islets, skeletal muscle*); *in vitro* (MIN6-B1 cells) • Insulin resistance • Lipotoxicity	Viability and function: • Increased β-cell proliferation and insulin secretion capacity via EV-mediated transfer of miR-16 (insulin resistant mice)	Implicated miRNA or biomimetic may have therapeutic potential for enhancing β-cell viability and insulin secretion capacity
**Hepatocyte/β-cell EV crosstalk**			
Fu et al. (49)	T2DM; *in vivo* (B6 mice, HPD vs SCD); *ex vivo* (mouse islets, primary hepatocytes*); *in vitro* (MIN6 cells) • Insulin resistance • Lipotoxicity	Viability: • Increased β-cell proliferation via EV transfer of miR-7218-5p (treatment with hepatocyte EVs from HFD mice compared treatment with EVs from SCD mice)	miR-7218-5p or biomimetic molecules as potential therapies to promote β-cell proliferation
**Adipocyte/β-cell EV crosstalk**			
*Gesmundo et al. (50)*	T2DM; ex vivo (human donor adipose tissue* and islets); in vitro (3T3-L1 adipocytes,* INS-1E cells, EndoC-βH3 cells) • *Pro-inflammatory cytokines* • *Hyperglycemia* • *Lipotoxicity*	*Viability and function:* • *Increased proliferation (treatment with EVs from cultured adipocytes, standard growth conditions)* • *Increased apoptosis (treatment with EVs from cultured adipocytes exposed to pro-inflammatory cytokines or palmitate)* • *Increased apoptosis, decreased insulin secretion (treatment with EVs from white adipose depots of obese donors)* • *Altered EV cargo miRNAs involved with insulin secretion, proliferation, apoptosis pathways*	*Implicated miRNAs may provide insights into potential miRNA-based therapeutics for T2DM*
**Unknown (circulating)/β-cell EV crosstalk**			
Garcia-Contreras et al. (51)	T1DM; *ex vivo* (serum* and islets from human donors)	Function: • Diminished second phase response of biphasic insulin secretion (treatment with serum EVs of T1DM subjects compared to treatment with EVs from control subjects) • Altered EV cargo miRNAs associated with β-cell function and with autoantibodies in subjects with T1DM	Implicated miRNAs may provide insights into potential miRNA-based therapeutics for T1DM
James-Allan et al. (52)	GDM; *in vivo* (female, non-pregnant B6 mice); *ex vivo* (mouse islets, plasma* from healthy non-pregnant, healthy pregnant, and pregnant-GDM women at 24–48 weeks gestation)	Function: • Elevated GSIS, serum insulin, and skeletal muscle insulin resistance (mice treated with small EVs from healthy pregnant women, compared to other treatment and control groups) • Diminished GSIS and impaired insulin signaling in skeletal muscle (mice treated with 4-day infusion of small EVs from pregnant women with GDM, compared to treatment with EVs from healthy pregnant women)	Insights into EV-mediated mechanisms of glucose homeostasis in pregnancy and GDM. Follow-up investigation of EV cargo shifts may lead to identification of early biomarkers for GDM
Li et al. (53)	T1DM and T2DM; *in vivo* (B6 WT and pre-miR-223 knockout mice; db/db mice); *in vitro* (MIN6 cells); intervention with previously validated EV cargo miR-223 • STZ-induced DM • HFD-induced obesity • Pro-inflammatory cytokines	Viability and function: • Impaired GSIS; decreased β-cell proliferation and increased apoptosis; altered expression of β-cell differentiation markers (knockout of miR-223 expression, *in vivo* and *in vitro*) • Enhanced GSIS; increased β-cell viability and rates of proliferation; decreased rates of apoptosis; normalization of β-cell differentiation markers (rescue of knockout phenotypes with miR-223 overexpression) • Mechanism of action involving Akt-mediated regulation of Foxo1 and Sox6 signaling pathways	miR-223 or biomimetic molecules as potential diabetes therapies to preserve adequate β-cell mass and insulin secretion
*Xie et al. (54)*	Obesity, T2DM; ex vivo (serum* from human donors); in vitro (unspecified β-cell line)	*Viability:* • *Decreased β-cell proliferation (treatment with EVs from obese/insulin-resistant donors, compared to EVs from lean donors)* • *Increased phosphorylation of NFkB, increased expression of CCL-2 (β-cells treated with EVs from obese donors vs. lean donors)* • *Decreased EV protein cargo of RICTOR, Omentin1 (EVs from obese donors vs. lean donors)*	*Identification of EV proteins that are potential therapeutic targets*

*Studies from the gray literature are distinguished from peer-reviewed, published studies by italic typeface. Asterisks indicate source of EVs for experimental use*.

*3T3-L1, mouse pre-adipocyte cell line; B6, C57BL/6 mouse strain; βTC-6, mouse β-cell line; Cip/Kip, CDK (cyclin-dependent kinase) interacting protein/Kinase inhibitory protein; Db/db, leptin-deficient mouse model of T2DM; EndoC-βH3, human β-cell line; HFD, high fat diet; HPD, high palmitate diet; IAPP, islet amyloid polypeptide; IFN-γ, interferon gamma; IL, interleukin; INS-1, rat insulinoma cell line; lncRNA, long noncoding RNA; MIN6, mouse insulinoma cell line; miR, miRNA, microRNA; MSC, mesenchymal stem cell; NCDase, neutral ceramidase; NOD, non-obese diabetic; NOR, non-obese diabetes resistant; PBMC, peripheral blood mononuclear cell; RAW264.7, mouse macrophage cell line; SCD, standard chow diet; SD, Sprague-Dawley rat; SCID, severe combined immunodeficient mouse; STZ, streptozotocin; Wnt, wingless-related integration site*.

### Description of Included Studies

More than half (n = 21) of the 31 selected studies were pertinent to the context of T1DM, with emphasis on the role of EVs in autoimmune (n = 10) and inflammatory (n = 13) processes during disease pathogenesis (Table 1). Another area of focus was stem cell-derived EVs and their potential as therapeutics to regenerate functional β-cell mass (n = 5). Attention was also given to alterations in EV crosstalk that occur in response to metabolic disruptions such as insulin resistance (n = 4), oxidative stress (n = 1), lipotoxicity (n = 6), and amyloid toxicity (n = 1). EV cargo molecules with potential activity in β-cells were identified or characterized in 24 studies, primarily miRNA (n = 16) and protein cargo (n = 8) (Table 2). Of the model systems employed in these studies, the majority included rodent models (n = 23) either exclusively (n = 13) or in combination with human tissue explants (n = 10). ex vivo human models were included in 17 studies, either alone (n = 4) or in combination with other model systems (n = 12). In vivo work (rodent models) was conducted in 15 studies (Table 3).

**Table 2:** EV cargo implicated in β-cell function/viability and methods of cargo characterization.

**First author, (ref)**	**EV class**	**Cargo type/implicated species**	**Methods**
Cantaluppi et al. (46)	Exosomes, microvesicles	miRNA (miR-126, miR-296)	In situ hybridization, qRT-PCR
Chen et al. (41)	Small EVs (exosomes)	miRNA (miR-21)	RNA-seq
Cianciaruso et al. (33)	Small EVs (exosomes)	Protein (GAD65, IA-2, proinsulin, calreticulin, Gp96, ORP150)	Liquid chromatography tandem mass spectrometry
Favaro et al. (43)	Small EVs (exosomes)	miRNA (miR-126) Protein (IFN-γ, IL-1, IL-17, IL-6)	Microarray
Figliolini et al. (47)	Exosomes, microvesicles	mRNA (eNOS, VEGFa, PDX-1, insulin, insulin receptor, IRS2, P13K, AKT2, GLUT4, G6Pase, GSK-3, PPARA, PPARG, PPARGC1A, PPARGC1B) miRNA (miR-27b, miR-126, miR-130, miR-296)	RT-PCR array, qRT-PCR, miRNA array
Fu et al. (49)	Small EVs (exosomes)	miRNA (miR07218-5p)	Microarray, RNA sequencing
Garcia-Contreras et al. (51)	Small EVs (exosomes)	miRNA (miR-16-5p, miR-302d-3p, miR-574-5p)	Microarray
Gesmundo et al. (50)	Small EVs (exosomes)	miRNA (unspecified)	RNA sequencing
Giri et al. (37)	Exosomes, microvesicles, apoptotic bodies	miRNA (miR-7a, miR-21, miR-29a, miR-29b, let-7b, let-7c) Protein (pro-insulin, insulin, IFNγ, IL-1β, IL-27, MCP-1, TNFα)	qRT-PCR, ELISA, cytometric bead assay
Guay et al. (24)	Small EVs (exosomes)	miRNA (miR-146a, miR-146b, miR-195, miR-290a-3p, miR-362-3p and miR497)	Microarray, qPCR
Guay et al. (34)	Small EVs (exosomes)	miRNA (miR-142-3p, miR-142-5p, miR-155)	qPCR miRNome profiling
Jalabert et al. (48)	Small EVs (exosomes)	miRNA (miR-16)	qRT-PCR array
Javeed et al. (30)	Exosomes, microvesicles	Protein (HLA-A, STAT1, INS, CPE)	Not described
Li et al. (45)	Small EVs (exosomes)	miRNA (let-7c-2-3p, miR-322-3p, miR-9a-5p, let-7a-1-3p, miR-27b-3p, miR-335, miR-322-3p)	RNA sequencing
Li et al. (53)	Previously validated small EV cargo	miRNA (miR-223)	
Ribeiro et al. (25)	Small EVs (exosomes)	Lipid composition, EV membrane ratio of lipids/proteins	HPLC with charged aerosol detection
Ruan et al. (26)	Small EVs (exosomes)	lncRNA (lncRNA-p3134)	Microarray
Salama et al. (35)	Small EVs (exosomes)	miRNA (miR-29b)	RT-qPCR
Sheng et al. (36)	Small EVs (exosomes)	Protein (GAD65)	Western blot
Sims et al. (31)	Small EVs (exosomes)	miRNA (miR-21)	Digital droplet PCR
Tang et al. (27)	Small EVs (exosomes)	Protein (NCDase)	HPLC, Western blot
Tsukita et al. (44)	Small EVs (exosomes)	miRNA (miR-106b-5p, miR-222-3p)	qPCR miRNome profiling
Xie et al. (54)	Small EVs (exosomes)	Protein (RICTOR, Omenin1)	ELISA, Western blot
Zhu et al. (29)	Small EVs (exosomes)	Protein (NCDase)	ELISA, HPLC

**Table 3:** Experimental models used in selected studies.

**First author, (ref)**	**In vivo**		**Ex vivo**				**In vitro**		
	**Mouse**	**Rat**	**Human**	**Mouse**	**Rat**	**Pig**	**Human**	**Mouse**	**Rat**
Bashratyan et al. (32)	**♦**							**♦**	
Cantaluppi et al. (46)	**♦**		**♦**				**♦**		
Chen et al. (41)			**♦**					**♦**	
Cianciaruso et al. (33)			**♦**	**♦**	**♦**				
Favaro et al. (43)			**♦**						
Figliolini et al. (47)			**♦**				**♦**		
Fu et al. (49)	**♦**			**♦**				**♦**	
Garcia-Contreras et al. (51)			**♦**						
Gesmundo et al. (50)			**♦**				**♦**	**♦**	**♦**
Giri et al. (37)				**♦**				**♦**	
Guay et al. (24)					**♦**			**♦**	**♦**
Guay et al. (34)	**♦**		**♦**						
Jalabert et al. (48)	**♦**		**♦**					**♦**	
James-Allan et al. (52)	**♦**		**♦**	**♦**					
Javeed et al. (30)				**♦**				**♦**	**♦**
Li et al. (45)		**♦**							
Li et al. (53)	**♦**			**♦**				**♦**	
Mahdipour et al. (38)		**♦**	**♦**						
Nojehdehi et al. (42)	**♦**			**♦**					
Ribeiro et al. (25)			**♦**				**♦**		
Ruan et al. (26)			**♦**	**♦**				**♦**	
Salama et al. (35)	**♦**			**♦**				**♦**	
Sheng et al. (36)	**♦**			**♦**				**♦**	
Sims et al. (31)				**♦**	**♦**				
Sun et al. (39)		**♦**	**♦**						
Sun et al. (28)	**♦**							**♦**	**♦**
Tan et al. (40)			**♦**			**♦**			
Tang et al. (27)									**♦**
Tsukita et al. (44)	**♦**		**♦**	**♦**					
Xie et al. (54)			**♦**						
Zhu et al. (29)					**♦**				**♦**

### EV Crosstalk Dynamics

The studies included in this synthesis shed light on the dynamics of EV-mediated crosstalk that affects β-cell function and/or viability, either directly through uptake of EVs by β-cells, or indirectly through uptake of EVs by immune cells and subsequent induction of an autoimmune response against β-cells (Table 1). Within the context of both T1DM and T2DM, cells and tissues of origin for this EV crosstalk included β-cells and other islet cells (24–31), immune cells (32–37), mesenchymal stem cells (38–43), bone marrow (44, 45), and vascular endothelium (46, 47). In T2DM, skeletal muscle (48), liver (49), and adipose cells (50) also secrete EVs that affect β-cells. Additionally, serum-derived EVs of unknown cellular origin affect insulin secretion dynamics in T1DM, T2DM, and GDM pancreatic islets (51–54).

#### Crosstalk With Other β-Cells and Islet Cells

A primary focus within this area of research was the impact of the islet microenvironment on EV cargo composition and the subsequent effects of those EVs on the viability and insulin-secretion capacity of the recipient β-cells (24–29, 31). Cargo of EVs from β-cells is altered in response to disruptions in the islet microenvironment. Such disruptions include exposure to inflammatory cytokines (24, 29, 31) and toxic lipid species (27, 28), persistent hyperglycemia (26), and the formation of amyloid aggregates (25). In the absence of noxious stimuli or with moderate disruptions to the islet microenvironment such as low-level inflammation or hyperglycemia, small EVs transfer regulatory molecules among cells within islets in order to coordinate insulin production and secretion and preserve β-cell viability (26–29). These regulatory factors include non-coding RNA species such as lncRNA-p3134 (55) and the enzyme neutral ceramidase (NCDase) (27, 29).

Injection of small EVs derived from the mouse MIN6 and rat INS-1 β-cell lines improved islet insulin content and glycemic control in mouse models of T1DM induced by streptozotocin (28). This treatment also increased angiogenesis within islets and diminished macrophage infiltration of islets (28). Cargo shifts and mechanisms for these effects were not investigated in this report, and further study of this phenomenon is merited.

Conditions of insulin resistance and persistent hyperglycemia in T2DM induced EV enrichment with lncRNA-p3134, which promoted β-cell adaptation to peripheral insulin resistance (26). Elevated EV cargo of lncRNA-p3134 was first observed in serum of human subjects with T2DM, compared to serum EVs from healthy subjects. This lncRNA was then identified as cargo of islet-derived EVs. Overexpression of lncRNA-p3134 in MIN6 cells and a mouse model of T2DM promotes compensatory insulin secretion by increasing both the amount of insulin produced and the rate of secretion, and this EV crosstalk within islets serves to amplify the adaptive response.

Small EVs released by mouse and rat β-cell lines were implicated in cellular responses to free fatty acid exposures such as palmitate (27, 28). EVs isolated from conditioned culture medium of β-cell lines, maintained under standard growth conditions, were protective against apoptosis when added to cultures of palmitate-exposed MIN6 and INS-1 β-cell lines (27, 28). Small EVs contributed to the amelioration of palmitate-induced apoptosis through transfer of NCDase cargo to other β-cells in vitro (27).

Low levels of inflammatory cytokines in the cellular environment, such as might be present in T2DM or prior to onset of T1DM, induced an EV-mediated adaptive response in β-cells (29). Under these conditions, both the INS-1 β-cell line and ex vivo rat islets secreted small EVs enriched in NCDase, which is protective against cytokine-induced cell injury and apoptosis. These EVs transmitted the protective enzyme to other β-cells throughout the culture or islet, thus helping to enhance β-cell survival and preserve insulin secretion capacity. In contrast, exposure to high levels of inflammation led to diminished expression of NCDase within small EVs and to loss of the protective effect (29). β-cell-derived EVs instead transferred pro-apoptotic miRNAs (miR-21, miR-146a/b, miR-195, miR-290a-3p, miR-362-3p, and miR-497) to other β-cells (24, 31). In mouse and rat islets as well as MIN6 and INS-1 β-cell lines, exposure to high levels of inflammatory cytokines induced enrichment of small EVs with miRNAs that were associated with apoptotic signaling pathways (24, 31). These EVs served to propagate pro-apoptotic signals within islets, resulting in diminished β-cell mass. These EVs also induced cell death when transferred to cultures of previously healthy β-cells with no inflammatory exposures (24). Exposure of MIN6 and INS-1 β-cell lines to pro-inflammatory cytokines stimulated increased release of microvesicles and exosomes, with a significant shift to smaller EVs with characteristics of exosomes (30). These EVs from cytokine-exposed cells were also observed to have increased cargo of pro-inflammatory and diabetogenic proteins HLA-A, STAT1, and INS. ex vivo treatment of mouse islets with these EVs produced islet phenotypes consistent with β-cell failure, compared to control treatment. Alterations in phenotype included significant reductions in insulin synthesis and glucose-stimulated insulin secretion capacity, as well as increased expression of CXCL10, TLR1, and TLR4, further exacerbating inflammation (30).

Small EVs may also play a role in prevention of amyloid toxicity in metabolically healthy subjects (25). The hormone islet amyloid polypeptide (IAPP) is secreted by β-cells and helps regulate glucose homeostasis (25). In T2DM, these IAPP fibers cluster together within islets to form amyloid deposits, which are toxic to β-cells. In cultures of β-cells in which IAPP aggregation has been induced, the addition of small EVs isolated from metabolically healthy human donors prevented formation of aggregates. Although the mechanism is unclear, this effect was absent with addition of EVs isolated from islets of human donors with T2DM. Lipidomic and proteomic analysis of EV membranes revealed an increased ratio of membrane proteins to lipids in EVs from T2DM islets, compared to those from healthy donors. IAPP fibers may have an affinity for the exposed lipid surface of EVs from metabolically healthy donors, and the altered membrane composition of EVs from T2DM donors may interfere with this interaction. Further in vitro and in vivo studies will help elucidate the mechanisms of these phenomena.

#### Crosstalk With Immune Cells

There is accumulating evidence for involvement of EV crosstalk in the autoimmune destruction of β-cells that occurs in T1DM. β-cell and islet auto-antigens such as GAD65, glucagon, insulin, and islet antigen-2 are present in small EVs isolated from MIN6 and INS-1 cell lines and from ex vivo human and rodent islets (33). Mouse splenocytes treated with small EVs from cultured β-cells exhibit increased expression of pro-inflammatory cytokines, an effect attributed to EV cargo of miR-29b (35). Such β-cell derived EVs appear to be capable of provoking an autoimmune response through activation of B lymphocytes (32), T lymphocytes (36), and dendritic cells (33, 35). This activation of immune cells occurs not only in vivo in the mouse models of predisposition to T1DM (32, 35, 36), but also ex vivo in primary cultures of splenocytes or peripheral blood mononuclear cells from rodent models (33, 35). Furthermore, EVs secreted by T lymphocytes act upon recipient β-cells by inducing apoptosis through the action of cargo miRNAs 142-3p, 142-5p, and 155 (34).

Only one study relevant to crosstalk between β-cells and immune cells included use of large EVs, including microvesicles and apoptotic bodies (37). In this pre-print article, it is reported that exposure of MIN6 cells to even low concentrations of pro-inflammatory cytokines may stimulate alterations in the quantity of EVs released and also in the EV cargo. Treatment of MIN6 cells with pro-inflammatory cytokines induced an increase in the release of all EV types, including exosomes, microvesicles, and apoptotic bodies, compared to control-treated cells (37). EV cargo shifts include increased cargo of β-cell auto-antigens insulin and pro-insulin in small EVs and apoptotic bodies, as well as increased cargo of miRNAs capable of binding TLR7 and activating autoimmune processes in target cells (37). These EVs derived from cytokine-exposed β-cells activate both dendritic cells (treatment with small EVs or apoptotic bodies) and macrophages (treatment with small EVs, microvesicles, or apoptotic bodies) in vitro (37).

#### Crosstalk With Mesenchymal Stem Cells (MSC)

EVs derived from MSCs of healthy donors show promise as therapies to protect against β-cell injury due to autoimmune processes, inflammation, and oxidative stress (38–40, 42). MSCs for these studies were isolated from a variety of sources, including human menstrual blood (38), umbilical cord (39, 40), and adipose tissue (42). EVs released by MSCs derived from human menstrual blood were used as an experimental intervention in Wistar rats with streptozotocin-induced T1DM; treated rats exhibited regeneration of functional β-cell mass within islets (38). EVs from human umbilical cord MSCs were likewise capable of repopulating β-cells and reducing levels of inflammatory cytokines in rodent models of T2DM (39). ex vivo, in porcine islets, these MSC-derived EVs protect against cell injury that often occurs in transplanted islets due to hypoxia and oxidative stress (40). Similarly, when the mouse βTC-6 β-cell line is cultured in hypoxic conditions, treatment with human MSC-derived EVs reduces hypoxia-induced endoplasmic reticulum stress as well as rates of apoptosis (41). This protective effect appears to be attributable to cargo enrichment of miR-21 in MSC EVs (41). There is further evidence that EVs released from adipose-derived MSCs affect β-cell function and viability. These EVs suppressed autoimmune destruction of β-cells in an in vivo mouse model of T1DM. Treatment also decreased levels of pro-inflammatory cytokines and increased levels of anti-inflammatory cytokines (42). Contradictory evidence is present in the gray literature, suggesting that EVs isolated from human adipose MSCs enclose pro-inflammatory cargo (miR-126 and proteins IFN-γ, IL-1, IL-6, and IL-17) and increase expression of pro-inflammatory cytokines in human peripheral mononuclear blood cells from T1DM and T2DM donors (43). However, whereas high-quality methods of MSC isolation and EV extraction are described in the research report of Nojedehi et al. (42), details with regard to MSC methods in the conference abstract by Favaro et al. (43) are scant. Since there is abundant evidence in the research literature reporting the anti-inflammatory effects and cargo of EVs derived from adipose MSCs in other inflammatory disease contexts (56–59), it is probable that the findings reported by Favaro et al. are in error.

#### Crosstalk With Bone Marrow Cells

Similar to MSCs, bone marrow cells from healthy donors are another source of EVs that may have the capacity to regenerate β-cells and protect against cell injury. Small EVs derived from mouse bone marrow cells include cargo miRNAs miR-106b-5p and miR-222-3p, which promoted β-cell proliferation by interfering with expression of cell-cycle regulatory proteins (44). in vivo functional studies of the effects of these miRNAs showed that they improved glycemic control and increased islet β-cell mass in a mouse model of streptozotocin-induced T1DM (44). Further investigation of EV-mediated interactions between bone marrow and β-cells was prompted by observations that liraglutide therapy improved both glycemic control and osteoporosis in post-menopausal women with T2DM. This investigation revealed that, during liraglutide treatment, bone marrow EVs of ovariectomized rats are enriched in miR-322-3p and miR-335, which, respectively, promote cell proliferation and enhance insulin secretion when internalized by β-cells, (45).

#### Crosstalk With Vascular Endothelium

EV-mediated crosstalk between islet cells and vascular endothelium is relevant to maintenance or restoration of a healthy islet microenvironment in early stages of T1DM or T2DM, and it is of particular interest in the context of islet transplantation as treatment for T1DM. In addition to risk for immune rejection, another barrier to transplant success is hypoxic injury to grafted islets due to inadequate vascular supply. Two studies investigated crosstalk between β-cells or islets and vascular endothelium mediated by microvesicles and exosomes (46, 47). The first of these involved use of exosomes and microvesicles isolated from endothelial precursor cells (EPCs) (46). Outcomes for treatment of ex vivo human islets with EPC EVs included improvement in glucose-stimulated insulin secretion and decreased rates of β-cell apoptosis. These EPC EVs also conferred survival benefit to islet cells indirectly by stimulating angiogenesis in islet endothelial cells, thereby improving vascular supply of islets. This effect is attributable in part to two cargo miRNAs (miR-126 and miR-296) that promote angiogenesis. Similar results were observed in vivo with mice after islet xenografts with or without EPC EVs (46). The second of these studies further characterized EV crosstalk between β-cells and islet endothelial cells, using ex vivo human islets and primary cultures of islet endothelial cells (47). Analysis of islet EVs indicated predominance of β-cell-derived exosomes and microvesicles in the sample, and cargo analysis highlighted the presence of miRNAs that promote angiogenesis. Treatment of islet endothelial cell cultures with islet EVs increased expression of proteins involved with angiogenesis and resulted in increased formation of capillary-like structures. This crosstalk again enhances β-cell viability indirectly by improving islet vasculature (47).

#### Crosstalk With Skeletal Muscle

In insulin-resistant mice fed a high-fat diet, skeletal muscle tissue released EVs that are selectively loaded with a muscle-specific miRNA (miR-16), which promotes β-cell proliferation (48). The subsequent increase in the quantity of β-cells further enhanced insulin-secretion capacity sufficiently to overcome peripheral insulin resistance. These effects were also observed during in vitro and ex vivo study of miR-16 activity in MIN6 cell cultures and mouse islets.

#### Crosstalk With Hepatocytes

Small EVs released by hepatocytes appear to be similarly capable of promoting expansion of islet β-cell populations (49). Primary hepatocytes derived from mice fed a high-fat diet released EVs with reduced cargo of miRNAs that limit β-cell proliferation, compared to hepatocytes from mice fed a standard chow diet. In particular, miR-7218-5p cargo is diminished, resulting in increased expression of pro-proliferative CD74 in MIN6 cells in vitro.

#### Crosstalk With Adipocytes

Evidence for adipocyte crosstalk with β-cells is limited to a single conference abstract (50). Since low-level inflammation is often present in adipose depots of individuals with obesity and diabetes, this research group examined the effect of pro-inflammatory cytokine exposure on EV cargo of mouse 3T3-L1 adipocytes in culture, compared to untreated cells. These effects included differential expression in EVs of miRNAs (unspecified in the abstract) involved with maintenance of functional β-cell mass. EVs from cytokine-treated or untreated 3T3-L1 cells were applied to cultures of rat INS-1E β-cells and to ex vivo human islets. They found that EVs from untreated adipocytes protected β-cells from injury and cell death associated with exposures to pro-inflammatory cytokines or palmitate. However, EVs from cytokine-treated adipocytes promoted apoptosis in β-cells exposed to similar conditions. This group further used ex vivo adipose tissue from obese human donors to isolate EVs. Treatment of the human β-cell line EndoC-βH3 with these EVs resulted in impairment of insulin secretion and β-cell death. Because of insufficient detail provided in the study abstract, it is unknown what control treatment or conditions were used to evaluate these effects. This preliminary evidence suggests that small EVs from adipose tissue may affect β-cells during normal physiological conditions as well as obesity. EVs derived from healthy adipose tissue may help maintain adequate β-cell mass, whereas EVs from obese or otherwise unhealthy adipose tissue may have a deleterious effect on β-cells.

#### Crosstalk With Serum-Derived EVs (Unknown Tissue Origin)

Cargo analysis of circulating small EVs revealed alterations in cargo molecules in subjects with T1DM (51) and T2DM (26) compared to control donors. Plasma EVs of T1DM donors had differential expression of several miRNAs, including one associated with insulin synthesis pathways (miR-25-30) and another associated with development of auto-antibodies (miR-574) (51). Treatment of ex vivo human islets with these EVs resulted in altered biphasic insulin secretion, with decreased insulin secretion during the second phase, compared to islets treated with EVs from healthy subjects (51).

LncRNA-p3134 was enriched in serum EVs of subjects with T2DM without severe complications of the disease, compared to serum EVs from control subjects (26). In mouse β-cell lines, this lncRNA species enhanced insulin production and secretion, and it may play a role in the compensatory insulin secretion that occurs in vivo in response to peripheral insulin resistance. While lncRNA-p3134 has been detected in cargo of islet-derived small EVs, these are not likely to account for the significant serum elevation of lncRNA-p3134-enriched EVs (26). Other potential tissue origins for small EVs enriched in lncRNA-p3134 include adipose tissue and skeletal muscle, which also express this lncRNA (26) and which are known to hyper-secrete small EVs when metabolically stressed (48, 60).

Small EV cargo of miR-223 is diminished in serum of subjects with T2DM compared to healthy subjects (53). In vivo functional studies of the effect of miR-223 in mouse models of T1DM and T2DM indicate that this cargo molecule is involved in β-cell regulatory processes including proliferation, differentiation, and insulin secretion (53). Knockout of miR-223 expression in mice yields a metabolic phenotype of impaired glucose-stimulated insulin secretion, decreased rates of β-cell proliferation, and increased rates of β-cell apoptosis. β-cell differentiation markers are also dysregulated. The severity of this phenotype is worsened with high-fat diet in vivo, and it is rescued in cell culture models with overexpression of miR-223 (53).

EV protein cargo differences are apparent between serum-derived small EVs from obese, insulin-resistant subjects, compared to lean control donors (54). Compared to EVs from lean donors, EVs from obese donors have diminished cargo of the proteins RICTOR and omentin-1. Treatment of cultured β-cells with EVs from obese subjects induced a decrease in rates of β-cell proliferation and increased markers of inflammation, compared to control-treated cells (54).

In a study of the impact of circulating EVs on β-cell function in the context of GDM, cargo shifts were not characterized (52). However, differential effects were observed among treatment groups involving small EVs isolated from plasma of healthy, non-pregnant women and pregnant women with and without GDM (52). Approximately 20% of the total plasma EVs from healthy pregnant women appear to be derived from placental tissue, as evidenced by the presence of the marker placental alkaline phosphatase, and in subjects with GDM, the proportion of placental-derived plasma EVs was significantly higher (52). In this study, non-pregnant mice were administered continuous infusions of EVs from these comparison groups (healthy pregnant, pregnant/GDM, and healthy non-pregnant women), with the result that in mice infused with small EVs from healthy pregnant women exhibited enhanced glucose-stimulated insulin secretion, hyperinsulinemia, and insulin resistance. This response was attenuated in mice infused with EVs from women with GDM, and these mice had diminished insulin secretion and impaired insulin signaling in skeletal muscle (52).

## Discussion

**Figure 3:**
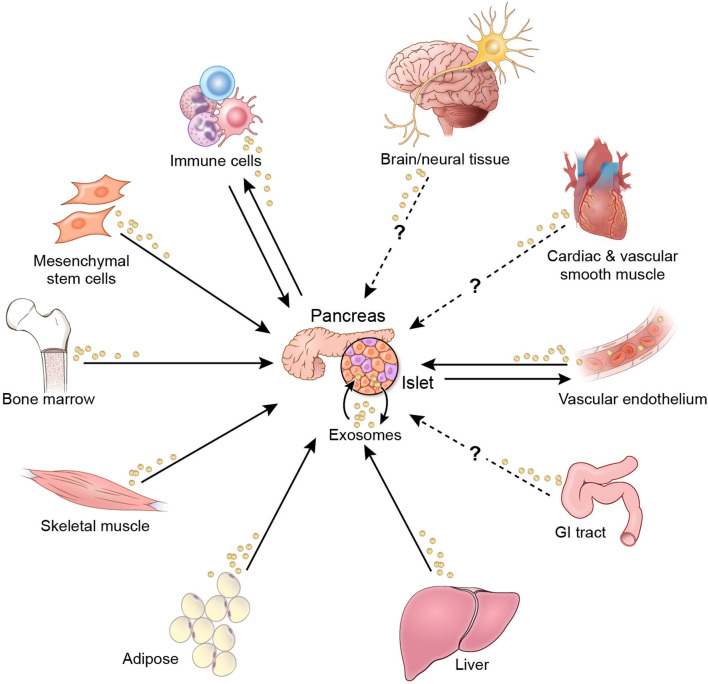
EV crosstalk affecting β-cell function and/or viability. Solid arrows indicate crosstalk for which there is current evidence. Dashed arrows indicate potential crosstalk axes.

The evidence reviewed herein provides insights into EV-mediated mechanisms for regulation of β-cell function and survival. In theory, EV crosstalk that affects β-cells may originate from organs and tissues throughout the body (Figure 3). Interorgan crosstalk, in the broader sense of secreted regulatory factors, occurs along each of the organ/tissue axes shown in Figure 3 (8, 9). Although there is not sufficient evidence to show EV-mediated crosstalk along all these axes, there is potential for such activity, since nearly all cells in the body produce and release EVs (11, 61–63). The research literature to date has focused primarily on EV crosstalk within islets and among β-cells, as well as on crosstalk axes involving immune cells and donor MSCs. However, little evidence is available that pertains to the impact of EVs from other organs and tissues involved in glucose homeostasis. Notably, the literature search yielded only one study each related to EV crosstalk originating from muscle, hepatic, and adipose tissue. In addition, more research is needed to understand the effect of anti-diabetic pharmacotherapies on EV cargo and crosstalk that affects β-cell function and viability.

A notable gap in this body of research is the relatively small number of studies pertinent to the disease contexts of T2DM and GDM. Although T2DM accounts for more than 90% of diabetes cases (1, 2), the majority of research related to the role of EVs in β-cell function and viability has focused on T1DM. The etiological context of 15 of the included studies were specific to T1DM, 9 were specific to T2DM, and an additional 6 studies had a shared context of both T1DM and T2DM (Table 1). Given the tremendous disease burden of T2DM in the United States and worldwide, there is a need for increased research activity on this topic that is pertinent to T2DM pathogenesis and therapies. In terms of β-cell outcomes in these studies, the predominant focus was on cell death, proliferation and insulin-secretion capacity, to the exclusion of the β-cell hypertrophy frequently observed in T2DM islets. Furthermore, this review identified only one experimental study specific to GDM (Table 1). Although GDM is usually transitory, women who experience GDM during a pregnancy are at 50% greater risk for developing T2DM subsequently, and children exposed to GDM *in utero* are also at significantly greater risk for developing T2DM than children not exposed to GDM (3). Thus, further attention should also be given to this topic. Other important additions to this body of research would be investigation of changes in EV crosstalk and cargoes over the course of diabetes progression. Many EV studies have focused on cargo shifts during disease onset and early disease stages as well as the end stages of β-cell failure. Generation of further evidence for the role of EV crosstalk over the full range of disease progression for T1DM, T2DM, and GDM would be beneficial. This should encompass aspects such as variations in EV crosstalk and cargoes due to disease severity, and development of diabetes complications. For example, it is probable that alterations in EV crosstalk affecting β-cells occur as peripheral insulin resistance progresses from mild to severe stages. Likewise, EV crosstalk and cargoes are likely to vary based on the severity and duration of autoimmune attacks in the development of T1DM. This has been examined to an extent in studies of the effects of cytokine exposures at varying concentrations (24, 29) but requires further investigation with regard to other stages and degrees of severity in T1DM autoimmune attacks.

An evaluation of this evidence in terms of the model systems used highlights a need for further validation of results obtained with *ex vivo* and *in vitro* systems, with subsequent *in vivo* work (Tables 1, 3). In addition, there is a need for further validation of findings from rodent studies, with follow-up studies using human tissues, cells, and donor islets. Other limitations of this research exist with regard to EV methods. The majority of these studies were conducted prior to publication of the Minimal Information for Studies of Extracellular Vesicles 2018 guidelines (MISEV 2018) (22). Consequently, many of these studies do not include or report all recommended components for functional studies using EVs. A summary of methods used in these studies for isolation and characterization of EV samples is provided in Table 4.

**Table 4:** Methods used in included studies for EV isolation, characterization, and validation of uptake.

**First author, (ref)**	**EV isolation (biofluid)**	**EV characterization**	**EV uptake assay**
Bashratyan et al. (32)	UC (CCM); P (serum)	TEM, MS, FC	
Cantaluppi et al. (46)	DC, UC (CCM)	TEM, FACS, PA	EV labeling
Chen et al. (41)	DC, UF, UC (CCM)	TEM, NTA, FC, WB	
Cianciaruso et al. (33)	DC, UC (CCM)	TEM, NTA, WB	EV labeling
Favaro et al. (43)	Not described	Not described	
Figliolini et al. (47)	DC, UC (CCM)	NTA, FACS, WB	EV labeling
Fu et al. (49)	DC, UC (CCM)	TEM, NTA, WB	EV labeling
Garcia-Contreras et al. (51)	DC, UC (plasma)	TEM, NTA, FC	
Gesmundo et al. (50)	Not described	Not described	
Giri et al. (37)	DC, UF, SEC (CCM)	Cryo-EM, TRPS, FC, WB	
Guay et al. (24)	DC, UC (CCM)	NTA, PA, WB	
Guay et al. (34)	DC, UC (CCM)	TEM, NTA	EV labeling
Jalabert et al. (48)	DC, UC (CCM)	NTA, PA	EV labeling
James-Allan et al. (52)	DC, DGC, UC (plasma)	TEM, F-NTA, WB	Non-endogenous cargo uptake
Javeed et al. (30)	Not described	Not described	
Li et al. (45)	DC, UC (CCM)	TEM, AFM, PA, WB	
Mahdipour et al. (38)	P, SEC (CCM)	SEM, AFM, WB, ELISA	EV labeling
Nojehdehi et al. (42)	DC, UC (CCM)	TEM, SEM, NTA, ζ	
Ribeiro et al. (25)	DC, P (CCM)	NTA, ζ, PA	
Ruan et al. (26)	P (serum)	Not described	
Salama et al. (35)	DC, UC (CCM)	NTA, PA	
Sheng et al. (36)	UF, UC (CCM)	TEM, MS, PA, WB	
Sims et al. (31)	Not described	Not described	
Sun et al. (39)	DC, UF (CCM)	TEM, NTA, PA, WB	EV labeling
Sun et al. (28)	DC, UC (CCM)	TEM, NTA, WB	
Tan et al. (40)	P (CCM)	TEM, NTA, PA, WB	
Tang et al. (27)	DC, UF, UC (CCM)	PA, WB	
Tsukita et al. (44)	P (serum, CCM)	Not described	
Xie et al. (54)	Not described	Not described	
Zhu et al. (29)	DC, UF, UC (CCM)	PA, WB	

*AFM, atomic force microscopy; CCM, conditioned culture medium; Cryo-EM, cryogenic electron microscopy; DC, differential centrifugation (for pre-clearance of cells and debris); DGC, density gradient centrifugation; ELISA, enzyme-linked immunosorbent assay; FACS, fluorescence-activated cell sorting; FC, flow cytometry with antibody-coated beads; F-NTA, fluorescence nanoparticle tracking analysis; MS, mass spectroscopy; NTA, nanoparticle tracking analysis; P, precipitation; PA, protein assay; SEC, size-exclusion chromatography; SEM, scanning electron microscopy; TEM, transmission electron microscopy; TRPS, tunable resistive pulse sensing; UC, ultracentrifugation; UF, ultrafiltration; WB, western blot; ζ, zeta potential*.

A limitation to many of these studies is that verification of EV biodistribution or uptake by the cell type of interest was conducted in only 9 of the 31 studies included in this review (Table 4). In 3 studies (38, 39, 48), fluorescently labeled EVs were administered by tail vein injection into rat or mouse models to investigate *in vivo* biodistribution, and EV uptake within organs was assessed *ex vivo* by fluorescence imaging systems. One other study assessed *in vivo* uptake of human plasma-derived EVs in mice by the presence in target cells of miRNAs unique to primates and not expressed in mice (52). Several other studies validated EV uptake *in vitro*, by adding labeled EVs to cells in culture, followed by confocal microscopy (33, 46, 49) or flow cytometry (34, 47) analysis of the cells to evaluate EV internalization. A single study investigated mechanisms of EV uptake by target cells, revealing the involvement of EV surface proteins ICAM1 and CD44 in the internalization of islet-derived EV by islet endothelial cells (47).

An additional limitation to these studies is mode and frequency of EV administration, which typically consists of discreet, timed doses of EVs. Evidence suggests that, *in vivo*, endogenous EVs are produced and released continuously by cells throughout the body (64–66). Moreover, pharmacokinetic studies suggest that EVs are cleared from circulation rapidly, largely due to cellular uptake, with half-life in circulation of 2 min to 5 h after injection *in vivo* (64, 67–69). Only one of the included studies addressed this limitation by engineering a system of continuous EV infusion at doses consistent with EV concentrations observed *in vivo* (52).

There are additional gaps across the literature regarding the classes of EVs investigated and the extent of EV cargo characterization. The majority of the studies reviewed here investigated the role of small EVs with characteristics of exosomes. Only 4 studies included microvesicles and only one study included apoptotic bodies in their experiments (Table 2). Of the 24 studies that characterized the effect of cargo molecules, the most frequently studied cargo was miRNA (15 studies), followed by protein (8 studies) (Table 2). There has been scant attention given to mRNA and lncRNA cargo as well as lipid content of EVs. Furthermore, few of these studies utilized methods that comprehensively characterized the cargo component of interest (i.e., miRNA, protein), as detailed in Table 2. More comprehensive characterization of EV cargo and composition, as well as investigation of corresponding effects on β-cells, may shed further light on diabetes pathogenesis and progression.

### Therapeutic Implications

This evidence elucidates mechanisms involved in T1DM, T2DM, and GDM pathogenesis. This in turn may aid in the development of more effective tools for monitoring β-cell function as well as novel, personalized interventions for prevention and treatment of diabetes. EV cargo alterations occur in response to metabolic disturbances such as persistent elevations in glucose, inflammatory cytokines, and toxic lipid species. Depending on the severity of the exposure, EV crosstalk may induce adaptive responses within pancreatic islets, including expansion of β-cell mass or increased insulin production and secretion. Crosstalk may also contribute to β-cell dysfunction and promote apoptosis.

The EV cargo implicated in diabetes pathogenesis in these studies provide insights into potential therapies and therapeutic targets (Tables 1, 2). Therapies involving EVs derived from stem cells offer hope for regeneration of functional islet β-cell mass. Although stem cell therapies for diabetes have been proposed, such regenerative therapies are limited by scarcity of suitable stem cells and by ethical considerations pertaining to use of embryonic stem cells (70, 71). Induced pluripotent stem cells and MSCs are additional sources of therapeutic progenitor cells; however, stem cell therapies are complicated by the risk of graft rejection and the risk for developing cancer (70–72). Because the therapeutic effect of stem cells is mediated in part by EVs, MSC EV-based therapies, such as those discussed in this review, represent a feasible alternative approach with reduced risk for adverse effects (38–40, 73).

The studies reviewed here identify bioactive EV cargo molecules that regulate β-cell viability and function (Tables 1, 2). Some of these proteins and non-coding RNAs may have potential for therapeutic use. Challenges to development of these molecules as therapeutics include enhancing their stability outside an EV membrane and targeting them to pancreatic islets. Stable oligonucleotides may be synthesized that mimic the activity of miRNA and other non-coding RNA species (44, 74). Similarly, antisense oligonucleotides can downregulate non-coding RNA species that impair β-cell function or survival (74, 75). However, targeted delivery of these potential therapeutics is necessary to minimize off-target effects (75). Engineered lipid nanovesicles are a possible delivery mechanism for therapeutic agents, but further evidence is required to understand mechanisms of EV targeting and delivery to specific cell types (76).

More thorough characterization of EV cargo in metabolically healthy and unhealthy subjects, as well as in those with different subtypes of diabetes, can help identify new therapeutic targets. Characterization of cargo shifts in islet-derived EVs throughout diabetes disease progression and subsequent validation of biomarkers may offer a non-invasive method of interrogating the health and functional status of β-cells, based on EV biomarkers. The effectiveness of EV biomarkers as diagnostic and monitoring tools for islet and β-cell function has been demonstrated recently in the context of islet transplantation both in animal models and human subjects (77–79). Biomarker discovery from this type of EV research may permit earlier detection of β-cell dysfunction and aid in development of more effective interventions prior to onset of glucose intolerance. Such evidence may also lead to advanced technologies for evaluating individual response to therapy, including real-time monitoring of β-cell function.

## Conclusion

There is noteworthy evidence for EV involvement in the progressive β-cell dysfunction and/or depletion that occur in T1DM, T2DM, and GDM. Further research that addresses the gaps described herein may provide a more thorough understanding of the complex interorgan crosstalk that governs glucose homeostasis and diabetes pathogenesis. This expansion of evidence and knowledge holds promise to decrease significantly the incidence of diabetes and to improve patient outcomes through earlier surveillance and personalized interventions.

## Author Contributions

SC: selected study design, assisted with literature searches, reviewed search results for eligibility, extracted data, directed figure design, composed manuscript. AL: assisted with selection of study design, designed and conducted literature searches, managed citations, and reviewed and edited final manuscript. AF: assisted with qualitative synthesis of results, reviewed and edited final manuscript. PJ: assisted with selection of study design, reviewed search results for eligibility, assisted with figure design, reviewed and edited manuscript.

## Conflict of Interest

The authors declare that the research was conducted in the absence of any commercial or financial relationships that could be construed as a potential conflict of interest.
