# Supplementary material for: The Role of Extracellular Vesicles in β-Cell Function and Viability: A Scoping Review
Source: Front Endocrinol (Lausanne). 2020 Jun 11;11:375. doi: 10.3389/fendo.2020.00375 (PMC7300279; doi:10.3389/fendo.2020.00375)
Supplement: Supplementary file 1 [file Data_Sheet_1.PDF]

Supplemental Methods 1

Scoping Review Protocol: Extracellular Vesicles and β-Cell Function/Viability

Review Question

What is the research evidence related to the effect of extracellular vesicles (EVs, consistent with characteristics of exosomes and microvesicles) on pancreatic β-cell function and/or viability, within the context of type 1, type 2, and gestational diabetes mellitus?

Objectives

- To characterize and map experimental research evidence related to the research question, including:
    - Focus areas of the research evidence, including diabetes type, aspects of disease etiology, and disease stage.
    - Model systems used to study the effect of EVs or their cargo on β-cells.
    - Organs and tissue types implicated in EV crosstalk that affects β-cells.
    - EV cargo molecules implicated in β-cell function and affected pathways in β-cells; associated insights into pathogenesis/disease mechanisms.
- To identify and describe gaps in the evidence.
- To discuss implications for diabetes therapy.

Inclusion and Exclusion Criteria

Inclusion criteria:

- Publication in the English language
- Original research studies
- Experimental research design
- Experimental intervention involving EVs (with characteristics consistent with exosomes and microvesicles) or validated small EV cargo
- Outcome measure of β-cell function and/or viability
    - i.e., β-cell apoptosis and/or proliferation; glucose-stimulated insulin secretion assay; serum insulin and serum glucose; homeostasis model assessment of β-cell function (HOMA-B)
- Disease context of T1DM or T2DM.

Exclusion criteria:

- Manuscript not available in the English language
- Non-experimental studies
- Reviews, editorials, opinion articles, and letters
- Studies focusing on circulating factors (such as proteins, mRNA, non-coding RNA, lipid species) that are not validated EV cargo.
- Studies focusing primarily on EVs other than exosomes or microvesicles (i.e.,

apoptotic bodies).
- Studies using biomimetic/engineered nanovesicles rather than naturally occurring EVs.
- Disease context other than T1DM, T2DM, or gestational diabetes (i.e., exclusion of pancreatic cancer, and pancreatic cancer-induced diabetes)

Databases

Cochrane Library, Embase, ProQuest Dissertations & Theses Global, PubMed/MEDLINE, Scopus, and Web of Science

Types of evidence

This scoping review will consider experimental research including published/peer-reviewed studies, as well as grey literature such as unpublished dissertations and abstracts/conference proceedings.

Search terms

The following search terms and variations thereof, including MeSH terms, will be used to conduct database searches:

> exosome, extracellular vesicle, microvesicle, nanovesicle, insulin-secreting cells, pancreatic β-cell, β-cell function, diabetes mellitus type 1, diabetes mellitus type 2, prediabetes, insulin resistance, glucose intolerance, obesity, and metabolic syndrome.

Review methodology:

Database searches will be conducted by AAL, who will also screen the search results for duplicate results and organize the results in an EndNote library. SC will conduct searches of grey literature including dissertations and theses, as well as manual searches of bibliographies. Unique results will be independently screened by S.C. and P.V.J. for inclusion and exclusion criteria. A.F.F. will arbitrate any disagreements with regard to inclusion/exclusion of studies.

Data mapping/charting:

For each selected study, data and key concepts will be extracted using a table that includes the following:

- Author(s) and year of publication
- Model system
- Disease context, physiological conditions
- EV crosstalk dynamics
- Cargo molecules implicated
- β-cell outcome measures of viability and/or function
