# Supplementary material for: The Role of Extracellular Vesicles in β-Cell Function and Viability: A Scoping Review
Source: Front Endocrinol (Lausanne). 2020 Jun 11;11:375. doi: 10.3389/fendo.2020.00375 (PMC7300279; doi:10.3389/fendo.2020.00375)
Supplement: Supplementary file 2 [file Data_Sheet_2.PDF]

Supplemental Methods 2

Table S1. Detailed search strategies used for electronic database searches.

Database	Search terms
Cochrane Library	#1: (exosome OR exosomes OR exosomal OR exososomal OR exsosomal OR "extracellular vesicle" OR "extracellular vesicles" OR nanovesicle OR nanovesicles OR microvesicle OR microvesicles OR “micro vesicle” OR “micro vesicles” OR “micro vesicular” OR “nano vesicles” OR “nano vesicle” OR “nano vesicular” OR “extra cellular vesicle” OR “extra cellular vesicles”):ti,ab,kw<br><br>#2: (“insulin secretion” OR “Insulin secreting” OR “insulin producing cell” OR “insulin producing cells” OR “pancreatic beta cell” OR “pancreatic beta cells” OR “pancreatic β cell” OR “pancreatic β cells” OR “pancreas islet beta” OR “pancreatic islet beta” OR “pancreas beta cell” OR “pancreas beta cells” OR “islet beta cell” OR “Islet beta cells” OR “beta islet cell” OR “beta islet cells” OR “beta cell dysfunction” OR “beta cell dysfunctions” OR “beta cell function” OR “beta cell functions” OR “β cell function” OR “β cell functions” OR “pancreatic β cell” OR “pancreatic β cells”):ti,ab,kw<br><br>#3: [mh "insulin-secreting cells"] OR [mh "Insulin Secretion"]<br><br>#4: ("type 2 diabetes" OR "adult onset diabetes" OR "type 1 diabetes" OR "autoimmune diabetes" OR diabetes OR diabetic OR diabetics OR "pre diabetes" OR prediabetes OR prediabetic OR prediabetics OR “pre diabetic” OR “pre diabetics” OR "insulin resistance" OR "insulin resistant" OR "glucose intolerance" OR "glucose intolerances" OR "glucose intolerant" OR "latent diabetes" OR "glucose tolerance impairment" OR "chemical diabetes" OR "impaired glucose tolerance" OR "borderline diabetes" OR “metabolic syndrome” OR “metabolic syndromes” OR “metabolic x syndrome” OR obesity OR obese OR overweight OR “adipose tissue hyperplasia” OR adiposity):ti,ab,kw<br><br>#5: [mh "Prediabetic State"] OR [mh "Diabetes Mellitus"] OR [mh “Glucose Intolerance”] OR [mh "diabetes mellitus, type 2"] OR [mh “diabetes mellitus, type 1”]<br><br>#6: #2 OR #3

	#7: #4 OR #5<br><br>#8: #1 AND #6 AND #7 = 0
Embase	(exosome:ti,ab OR exosomes:ti,ab OR exosomal:ti,ab OR exososomal OR exsosomal OR "extracellular vesicle":ti,ab OR "extracellular vesicles":ti,ab OR nanovesicle:ti,ab OR nanovesicles:ti,ab OR microvesicle:ti,ab OR microvesicles:ti,ab OR “micro vesicle”:ti,ab OR “micro vesicles”:ti,ab OR “micro vesicular”:ti,ab OR “nano vesicles”:ti,ab OR “nano vesicle”:ti,ab OR “nano vesicular”:ti,ab OR "extra cellular vesicle":ti,ab OR "extra cellular vesicles":ti,ab OR 'exosome'/exp OR 'nanovesicle'/exp) AND ('pancreas islet beta cell'/exp OR 'insulin release'/exp OR 'beta cell dysfunction'/exp OR 'insulin secretion':ab,ti OR 'insulin secreting':ab,ti OR 'insulin producing cell':ab,ti OR 'insulin producing cells':ab,ti OR 'pancreatic beta cell':ab,ti OR 'pancreatic beta cells':ab,ti OR 'pancreatic β cell':ab,ti OR 'pancreatic β cells':ab,ti OR 'pancreas beta cell':ab,ti OR 'pancreas beta cells':ab,ti OR 'pancreas islet beta':ab,ti OR "pancreatic islet beta":ab,ti OR 'islet beta cell':ab,ti OR 'islet beta cells':ab,ti OR 'beta islet cell':ab,ti OR 'beta islet cells':ab,ti OR 'beta cell dysfunction':ab,ti OR 'beta cell dysfunctions':ab,ti OR 'beta cell function':ab,ti OR 'beta cell functions':ab,ti OR 'β cell function':ab,ti OR 'β cell functions':ab,ti) AND ('diabetes mellitus'/exp OR 'impaired glucose tolerance'/exp OR 'insulin dependent diabetes mellitus'/exp OR 'non insulin dependent diabetes mellitus'/exp OR 'autoimmune diabetes'/exp OR 'impaired glucose tolerance'/exp OR 'insulin resistance'/exp OR 'glucose tolerance'/exp OR diabetes:ti,ab OR diabetic:ti,ab OR diabetics:ti,ab OR 'type 2 diabetes':ab,ti OR 'adult onset diabetes':ab,ti OR 'type 1 diabetes':ab,ti OR 'autoimmune diabetes':ab,ti OR 'diabetes mellitus':ab,ti OR 'pre diabetes':ab,ti OR prediabetes:ab,ti OR prediabetics:ab,ti OR prediabetic:ab,ti OR “pre diabetic”:ab,ti OR “pre diabetics”:ab,ti OR 'insulin resistance':ab,ti OR 'insulin resistant':ab,ti OR 'glucose intolerance':ab,ti OR 'glucose intolerances':ab,ti OR 'glucose intolerant':ab,ti OR 'glucose tolerance impairments':ab,ti OR 'glucose tolerance impairment':ab,ti OR 'chemical diabetes':ab,ti OR 'latent diabetes':ab,ti OR 'impaired glucose tolerance':ab,ti OR 'borderline diabetes':ab,ti OR “metabolic syndrome”:ti,ab OR “metabolic syndromes”:ti,ab OR “metabolic x syndrome”:ti,ab OR obesity:ti,ab OR obese:ti,ab OR overweight:ti,ab OR “adipose tissue hyperplasia”:ti,ab

	OR adiposity:ti,ab OR 'metabolic syndrome X'/exp OR 'obesity'/exp OR 'insulin resistance'/exp)
PubMed/MEDLINE	(exosome[tiab] OR exosomes[tiab] OR exosomal[tiab] OR exososomal[tiab] OR exsosomal[tiab] OR "extracellular vesicle"[tiab] OR "extracellular vesicles"[tiab] OR nanovesicle[tiab] OR nanovesicles[tiab] OR microvesicle[tiab] OR microvesicles[tiab] OR "extra cellular vesicle"[tiab] OR "extra cellular vesicles"[tiab] OR “micro vesicle”[tiab] OR “micro vesicles”[tiab] OR “micro vesicular”[tiab] OR “nano vesicles”[tiab] OR “nano vesicle”[tiab] OR “nano vesicular”[tiab]) AND ("insulin-secreting cells"[MeSH] OR "Insulin Secretion"[MeSH] OR "insulin secretion"[tiab] OR "Insulin secreting"[tiab] OR "insulin producing cell"[tiab] OR "insulin producing cells"[tiab] OR "pancreatic beta cell"[tiab] OR "pancreatic beta cells"[tiab] OR "pancreas beta cell"[tiab] OR "pancreas beta cells"[tiab] OR "islet beta cell"[tiab] OR "Islet beta cells"[tiab] OR "beta islet cell"[tiab] OR "beta islet cells"[tiab] OR "pancreas islet beta"[tiab] OR "pancreatic islet beta"[tiab] OR "beta cell dysfunction"[tiab] OR "beta cell dysfunctions"[tiab] OR "beta cell function"[tiab] OR "beta cell functions"[tiab]) AND ("Diabetes Mellitus"[Mesh] OR diabetes[tw] OR "diabetes mellitus, type 2"[MeSH] OR "type 2 diabetes"[tiab] OR "adult onset diabetes"[tiab] OR "diabetes mellitus, type 1"[MeSH] OR "type 1 diabetes"[tiab] OR "autoimmune diabetes"[tiab] OR diabetic[tiab] OR diabetics[tiab] OR "Prediabetic State"[Mesh] OR "pre diabetes"[tiab] OR prediabetes[tiab] OR prediabetic[tiab] OR prediabetics[tiab] OR “pre diabetic”[tiab] OR “pre diabetics”[tiab] OR "insulin resistance"[tiab] OR "insulin resistant"[tiab] OR "Glucose Intolerance"[Mesh] OR "glucose intolerance"[tiab] OR "glucose intolerances"[tiab] OR "glucose intolerant"[tiab] OR "glucose tolerance impairment"[tiab] OR "impaired glucose tolerance"[tiab] OR "chemical diabetes"[tiab] OR "latent diabetes"[tiab] OR "borderline diabetes"[tiab] OR “metabolic syndrome”[tiab] OR “metabolic syndromes”[tiab] OR “metabolic x syndrome”[tiab] OR obesity[tiab] OR obese[tiab] OR overweight[tiab] OR "adipose tissue hyperplasia"[tiab] OR adiposity[tiab] OR "Metabolic Syndrome"[Mesh] OR "Insulin Resistance"[Mesh] OR "Obesity"[Mesh]) AND English[lang]
Scopus	TITLE-ABS-KEY ((exosome OR exosomes OR exosomal OR exososomal OR exsosomal OR {extracellular vesicle} OR {extracellular vesicles} OR nanovesicle OR nanovesicles OR microvesicle OR microvesicles OR {extra cellular vesicle} OR {extra cellular vesicles}

	OR {micro vesicle} OR {micro vesicles} OR {micro vesicular} OR {nano vesicles} OR {nano vesicle} OR {nano vesicular}) AND ({insulin secreting cell} OR {insulin secreting cells} OR {insulin secretion} OR {Insulin secreting} OR {insulin producing cell} OR {insulin producing cells} OR {pancreatic beta cell} OR {pancreatic beta cells} OR {pancreas beta cell} OR {pancreas beta cells} OR {islet beta cell} OR {Islet beta cells} OR {beta islet cell} OR {beta islet cells} OR {pancreatic islet beta} OR {pancreas islet beta} OR {beta cell dysfunction} OR {beta cell dysfunctions} OR {beta cell function} OR {beta cell functions} OR {β cell function} OR {β cell functions} OR {pancreatic β cell} OR {pancreatic β cells}) AND ({type 2 diabetes} OR {adult onset diabetes} OR {type 1 diabetes} OR {autoimmune diabetes} OR diabetes OR diabetic OR diabetics OR {pre diabetes} OR prediabetes OR prediabetic OR prediabetics OR {pre diabetic} OR {pre diabetics} OR {insulin resistance} OR {insulin resistant} OR {glucose intolerance} OR {glucose intolerances} OR {glucose intolerant} OR {latent diabetes} OR {glucose tolerance impairment} OR {chemical diabetes} OR {impaired glucose tolerance} OR {borderline diabetes} OR {metabolic syndrome} OR {metabolic syndromes} OR {metabolic x syndrome} OR obesity OR obese OR overweight OR {adipose tissue hyperplasia} OR adiposity))
Web of Science	TS=((exosome OR exosomes OR exosomal OR exososomal OR exsosomal OR "extracellular vesicle" OR "extracellular vesicles" OR nanovesicle OR nanovesicles OR microvesicle OR microvesicles OR "extra cellular vesicle" OR "extra cellular vesicles" OR “micro vesicle” OR “micro vesicles” OR “micro vesicular” OR “nano vesicles” OR “nano vesicle” OR “nano vesicular”) AND ("insulin secretion" OR "Insulin secreting" OR "insulin producing cell" OR "insulin producing cells" OR "pancreatic beta cell" OR "pancreatic beta cells" OR "pancreas beta cell" OR "pancreas beta cells" OR “pancreatic islet beta” OR "pancreas islet beta" OR "islet beta cell" OR "Islet beta cells" OR "beta islet cell" OR "beta islet cells" OR "beta cell dysfunction" OR "beta cell dysfunctions" OR "beta cell function" OR "beta cell functions" OR “β cell function” OR “β cell functions” OR “pancreatic β cell” OR “pancreatic β cells”) AND ("type 2 diabetes" OR "adult onset diabetes" OR "type 1 diabetes" OR "autoimmune diabetes" OR diabetes OR diabetic OR diabetics OR "pre diabetes" OR prediabetes OR prediabetic OR prediabetics OR “pre diabetic” OR “pre diabetics” OR "insulin resistance" OR "insulin resistant" OR "glucose intolerance" OR "glucose

	intolerances" OR "glucose intolerant" OR "glucose tolerance impairment" OR "impaired glucose tolerance" OR "chemical diabetes" OR "borderline diabetes" OR "latent diabetes" OR “metabolic syndrome” OR “metabolic syndromes” OR “metabolic x syndrome” OR obesity OR obese OR overweight OR “adipose tissue hyperplasia” OR adiposity))
